# Global DNA cytosine methylation variation in *Spartina alterniflora* at North Inlet, SC

**DOI:** 10.1371/journal.pone.0203230

**Published:** 2018-09-10

**Authors:** Trenton Agrelius, Jeffry L. Dudycha, James T. Morris

**Affiliations:** 1 Belle W. Baruch Institute for Marine and Coastal Sciences, University of South Carolina, Columbia, South Carolina, United States of America; 2 Department of Biological Sciences, University of South Carolina, Columbia, South Carolina, United States of America; Louisiana State University, UNITED STATES

## Abstract

*Spartina alterniflora*, marsh grass, is a vegetative apomicticly-reproducing halophyte native to marshes along the east coast of the United States and invasive across the world. *S*. *alterniflora* provides many ecosystem services including, but not limited to, water filtration, habitats for invertebrates, and sediment retention. Widespread diebacks of longstanding marsh grass colonies launched extensive investigations into probable mechanisms leading to patchy diebacks. There is still current debate as to the causes of a marsh dieback but environmental stress is acknowledged as a constant. Spatial epigenetic variation could contribute to variation of stress susceptibility, but the scale and structure of epigenetic variation is unknown. The current study investigates patterns of epigenetic variation in a natural population of *S*. *alterniflora*. This study examines variation of global DNA methylation within and among clones of the marsh grass *Spartina alterniflora* using an ELISA-like microplate reaction and observed significant heterogeneity of global DNA methylation within and among clones of *S*. *alterniflora* across the North Inlet basin, as well as significant differences of global methylation between adults and sexually produced seedlings. The present study also characterized differences for plants in a section of the population that experienced an acute marsh dieback in the year 2001 and have subsequently recolonized, finding a significant positive correlation between cytosine methylation and time period of colonization. The significant heterogeneity of global DNA methylation both within and among clones observed within this natural population of *S*. *alterniflora* and potential impacts from hypersaline environments at North Inlet suggests the need for more in-depth epigenetic studies to fully understand DNA methylation within an ecological context. Future studies should consider the effects of varying saline conditions on both global DNA and gene specific methylation.

## Introduction

Atlantic and Gulf Coast saltmarshes gained considerable notoriety from large-scale unexplained dieback events of the smooth cordgrass *Spartina alterniflora* in the year 2000. Investigations into causes of the 100,000 hectare (ha) dieback of *S*. *alterniflora* [1] within the Mississippi deltaic plain alone ensued over the following decade but no singular mechanism could be identified. The loss in the year 2000 was so alarming that it prompted the Louisiana governor to declare a state of emergency across the state [2]. Such extreme measures were warranted due to the many ecosystem services *S*. *alterniflora* provides including the filtration of water, the creation of habits for invertebrates, and the trapping of sediment through extensive underground networks of rhizome and root matter. Trapping sediments and other solids provides an effective buffer that reduces costal erosion and shoreline scouring, which in turn promotes overall saltmarsh health and coastal economies. Reported losses of Louisiana coastal marshes have been estimated at 65–91 km^2^ annually, resulting in the creation of bare mudflat and open water [3] representing 80% of the coastal wetland loss nationally [4].

*S*. *alterniflora* is a low-intertidal plant species that has demonstrated superior growth characteristics for marsh and coastline restoration projects making this species the primary choice of many federal and state agencies for vegetative restoration projects. The underground network of rhizomes, underground stems, and root matter can tolerate fluctuating water levels, wide ranges of salinity concentrations, and various soil types. There is much dispute regarding the cause of the dieback events but environmental stress is one the of acknowledged constants across sites. Little is known about what cultivates *S*. *alterniflora’s* susceptibility to stressors beyond the proposed abiotic stressors of drought and subsequent increased salinity conditions. Dieback events in Louisiana and elsewhere occurred only in spatially limited areas leaving populations of *Juncus roemerianus*, *S*. *patens*, and *Avicennia gerimans* (mangroves) largely unaffected further confounding the mystery [1]. It is possible that epigenetic variation within *S*. *alterniflora* plants and its response to environmental stimuli could explain part of the mechanism behind the dieback phenomena.

Epigenetics is defined as the study of heritable changes in gene expression and function that cannot be explained by alterations in the nucleotide sequence of DNA [5,6]. Altering gene expression and function is achieved mechanistically through reasonably well-defined molecular processes that either activate, reduce, or shut off the activity of genes. Presently, there is ample evidence of three epigenetic mechanisms—DNA methylation, remodeling of chromatin, and small RNA mediated regulatory processes—cooperatively working in concert to achieve altered gene expression and functionality [7–9]. DNA methylation is a process in which a methyl group (CH_3_) is added to the 5’-position on a cytosine nucleotide, resulting in 5’-methylcytosine [10]. This particular mechanism is perhaps the best studied and understood epigenetic process [11].

DNA methylation is crucial for stable gene regulation and silencing of transposable elements (TE) and repetitive elements (RE) in the plant genome [12]. The sequence context of methylation can vary between both dinucleotide (CpG) and trinucleotide (CpHpG and CpHpH, where H can represent either A, T, or C) sequences, being maintained by separate methyltransferase families [12–14]. Methylation of the CpG sequence is typically seen in promoter regions and acts as a regulator of gene expression, while methylation of either of the trinucleotide sequences is more closely associated with transposon inactivation [15,16]. In either context, DNA methylation has typically been characterized as a developmental process for all eukaryotic organisms undergoing dynamic changes in the number and position of methyl groups during various processes including embryogenesis, gametogenesis [17,18], cell proliferation, and cell differentiation within the meristematic regions of plants [19] and in response to both abiotic and biotic stressors [20–23].

The stability of epigenetic modifications within the cell, such as DNA methylation, has been extensively studied [24–28] with more recent discoveries of inheritance of the modifications across generations [5]. Environmentally-induced epigenetic variation and inheritance has been documented in genetically identical ramets [29], salinity stressed dandelions [22], and even through the meiotic inheritance of epialleles differing epigenetically but not in nucleotide sequence [30]. Higher levels of epigenetic variation have been seen in natural populations [31–34] and can be structured by not only genetic variation but also local environmental conditions. Studies using the clonally reproducing Japanese knotweed (*Fallopia japonica*) have shown habitat-specific phenotypes [35] and greater epigenetic rather than genetic variation within those habitat-specific phenotypes [32]. Substantial evidence suggests that epigenetic modifications may influence ecological processes at both the individual and population levels [23,32,36,37], but as Alonso et al. [38] pointed out, considerably more research on natural populations is needed before ecological significance of epigenetic variation can be accurately defined.

In the present study, global DNA cytosine methylation (5mC) within natural populations of the marsh grass *Spartina alterniflora*, a vegetative apomicticly-reproducing halophyte native to marshes along the east coast of the United States and invasive across the world [39–43], was examined. Expansion of this species occurs primarily through the propagation of vegetative clones from underground rhizomes, though brief stages of sexual reproduction can occur to establish new colonies. Some research has been reported in recent years to understand more on the epigenetic variation with *S*. *alterniflora*, particularly in reference to abiotic stressors [44,45] but still little is known about variation of methylation within and among clones of this species in natural populations. Epigenetics, more specifically DNA methylation, could be an important modulator with how organisms interact with the environment. Although there are other types of methylation, in this paper the term methylation refers exclusively to DNA methylation. This study sought to give an ecological context of an epigenetic modification by quantifying global DNA methylation within and among *S*. *alterniflora* plants at North Inlet. This was achieved by sampling 1) adult population across the basin, 2) distinct isolated genets, 3) known dieback locations, and 4) sexually produced seedlings.

## Materials and methods

### Study sites and sample collection

**Fig 1:**
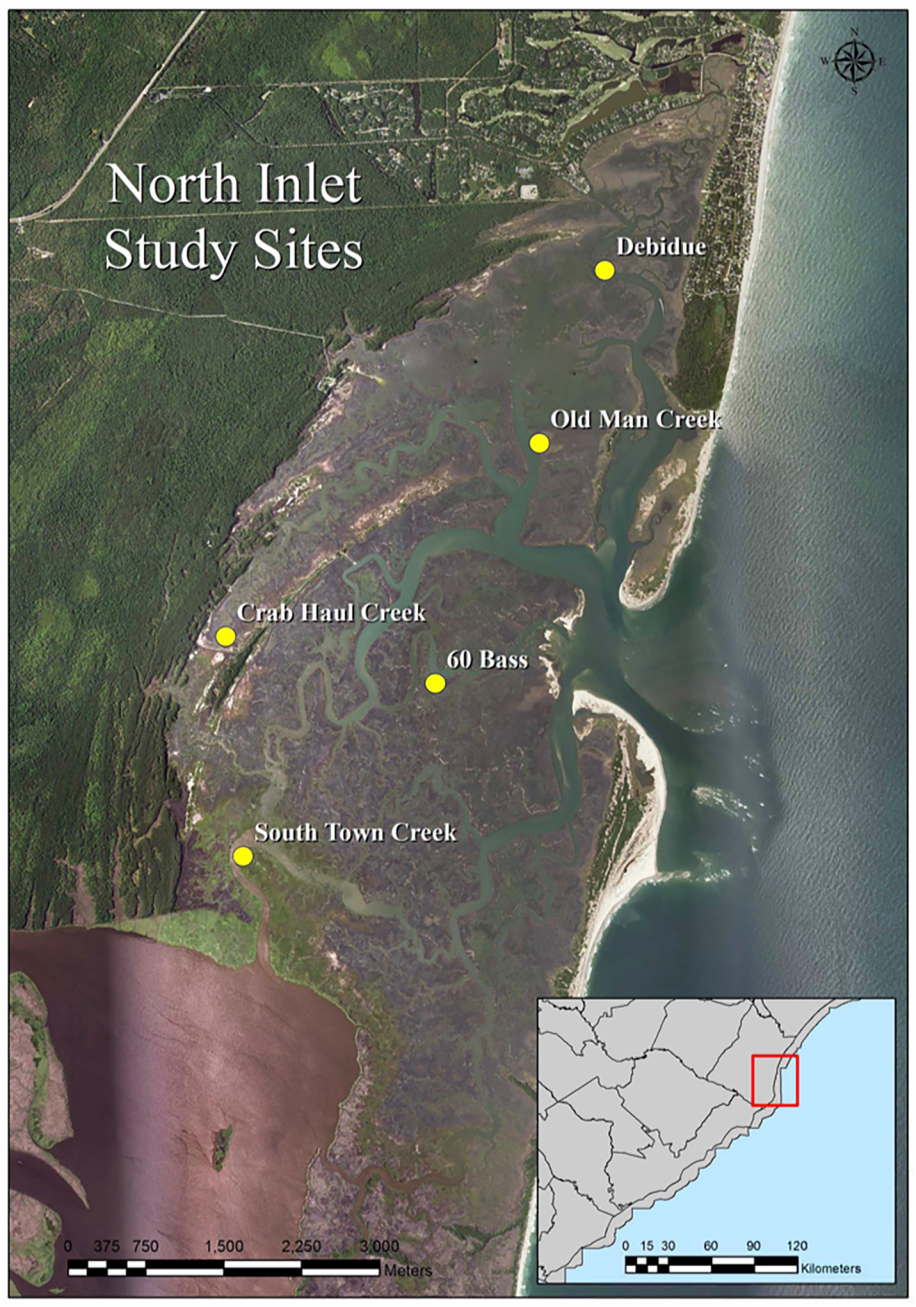
North Inlet, SC. Map of North Inlet outside of Georgetown, South Carolina with a cross section of the South Carolina coast taken from USGS National Map Viewer (http://viewer.nationalmap.gov/viewer/). Sampling locations have been marked with a yellow spot.

The study was conducted within the North Inlet-Winyah Bay National Estuarine Research Reserve System [33°19’ 37”N, 79°09’5”W] (Fig 1) located near Georgetown, South Carolina, USA. The bar-built estuary is dominated by *S*. *alterniflora*, covering approximately 19 km^2^ of the 33 km^2^ basin [46]. The reserve provides an opportunity to study the structure of variation of methylation by offering a range of plants varying in age, as well as identifiable isolated genet colonies, a documented acute marsh dieback (AMD) recolonization site [47], and sexually produced *S*. *alterniflora* seedlings.

In order to characterize clonal variation of methylation within *S*. *alterniflora* clones, leaf tissue from randomly selected stems across five sites in the North Inlet basin as shown in Fig 1 was collected. The sites included Crab Haul Creek [33°19’39”N, 79°12’21”W], South Town Creek [33°18’30”N, 79°12’15”W], Sixty Bass Creek [33°19’24”N, 79°11’02”W], Debidue Creek [33°21’30”N, 79°09’56”W], and Old Man Creek [33°20’40”N, 79°10’23”W]. Characterizing within and among clone variation requires the identification of isolated and independent *S*. *alterniflora* clones, which was not possible at most sites. The current study used Google Earth Historical Imagery photos [48] from 2013/14 to identify locations within North Inlet sparsely populated by *S*. *alterniflora* where potentially new genet colony expansion could occur. This study targeted four clearly identifiable isolated colonies for sampling within the Crab Haul Creek basin [33°20'23.36"N, 79°12'5.59"W]. Each colony was divided into two transects (North/South, East/West) establishing an intersection point which was assumed to be the oldest part of the colony. Along each transect, samples were taken at the clonal edge, midpoint from the edge to the center, and the center (n = 10/genet, 5 per transect).

Sixty Bass, a marsh island within North Inlet, experienced an AMD in 2001. As a result it became a well-documented study site for potential links between various hydrological variables, including porewater redox changes and hypersalinity within the root zone, and AMD [47]. Recolonization following this dieback provides three distinct age ranges within the Sixty Bass location: the oldest surviving healthy marsh that did not experience the dieback, the intermixed periphery surrounding the dieback epicenter which suffered moderate dieback causalities, and the dieback epicenter itself. Each region was randomly sampled (n = 10/region) and are subsequently referred to as Healthy Marsh, AMD Periphery, and AMD respectively (Morris and Sundberg unpublished data).

The South Town location was composed of a thick periphery of adult ramets surrounding a mudflat containing recently produced seedlings. The adult ramets selected for this study were apart of various asexual genets within the South Town location (n = 10) while the seedlings represent recent sexual reproduction (n = 10).

For each tissue collection in this study, the top fully-expanded leaf of each plant was used to minimize sampling bias due to developmental variation. Samples were taken in March 2014 and flash frozen in liquid nitrogen, ground in a fine powder and stored at -80°C. Total genomic DNA was extracted from each sample in duplicate using Qiagen DNeasy Plant Mini kits following the manufacturer’s specifications (100 ng of tissue per sample). Sample quality and quantity were estimated using the NanoDrop 2000 (NanoDrop Technologies Inc. Rockland, DE, USA), as well as ethidium bromide staining on agarose gels.

### Global methylation quantification

Global methylation of DNA isolated from frozen leaf tissue samples was quantified using the fluorometric Methylflash Methylated DNA Quantification Kits (EpiGentek) following the manufacturer’s instructions. Methylation status was detected using an ELISA-like 96-well microplate-based reaction and quantified fluorometrically using absorbance readings (relative fluorescence readings, RFU) at excitation 530 nm and emission 590 nm. Reported as a percentage, detected methylation level was calculated as a percentage of cytosine genomic content. To do this, a standard curve was generated with a positive control of known methylation that was provided in the kit. The standard curve ranged from 0.2 to 10.0 ng of the 50% methylated control, and was normalized by multiplying by a factor of 2 to 100%. Thus for a sample 5mC (ng) = (Sample RFU − negative control)/ (slope of standard curve x 2). Once a mass value was obtained, then the percentage of 5mC was calculated using 5mC (%) = (5mC(ng)/*S*) x 100, where *S* is the mass of the sample DNA. All samples used an input DNA concentration of 100 ng in the assay plate and were measured in triplicate to control for edge-effect errors prone to ELISA protocols; the mean of these technical replicates is reported. Internal positive (100% 5mC) and negative controls (0% 5mC) were also run in triplicate.

### Data analysis

Normality and homogeneity of variances were tested for. When either were violated non-parametric tests were used, each test is defined accordingly. Differences in methylation variation from each field site were identified using a Kruskal-Wallis one-way analysis and Games-Howell post-hoc test. A Pearson correlation was used to test for an association between age (position within genet) and methylation. A Spearman correlation test was used to identify variation within the AMD samples methylation and relative age of the marsh stand. Differences in methylation between the South Town sexual seedlings and asexual adults were identified using a Student’s t-test. All analyses were done using R 3.2.0 [49]. A p-value < .05 was considered statically significant. To evaluate measurement error, coefficient of variation (CV) values were calculated for the technical replicates of each biological sample, all biological replicates from each sampling location, and all biological samples from North Inlet.

## Results

**Fig 2:**
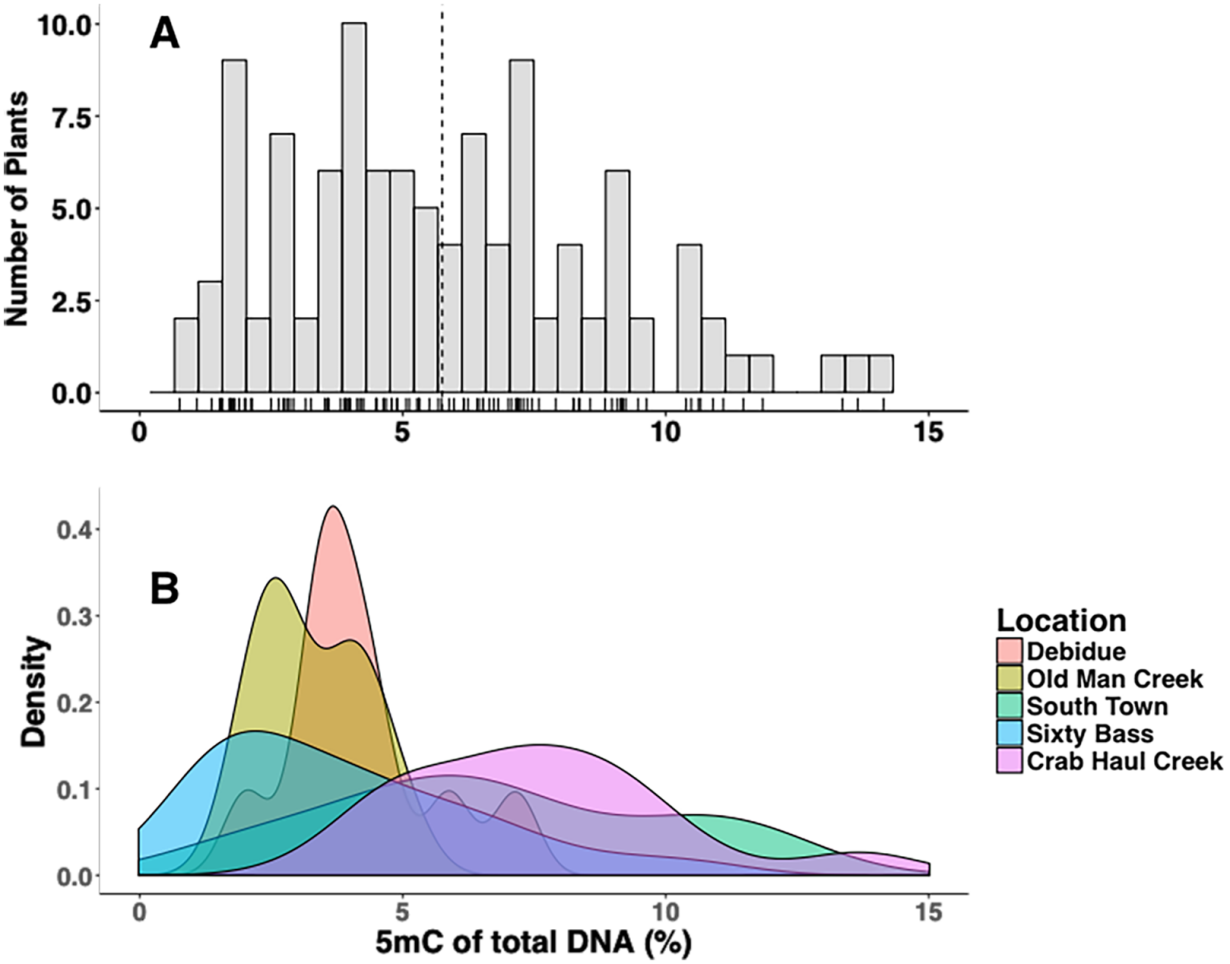
Methylation variation in *S*. *alterniflora* within and among sites at North Inlet, SC. **A**. frequency distribution of all methylation observations in this study. **B**. Frequency distribution of all methylation observations at each site.

**Fig 3:**
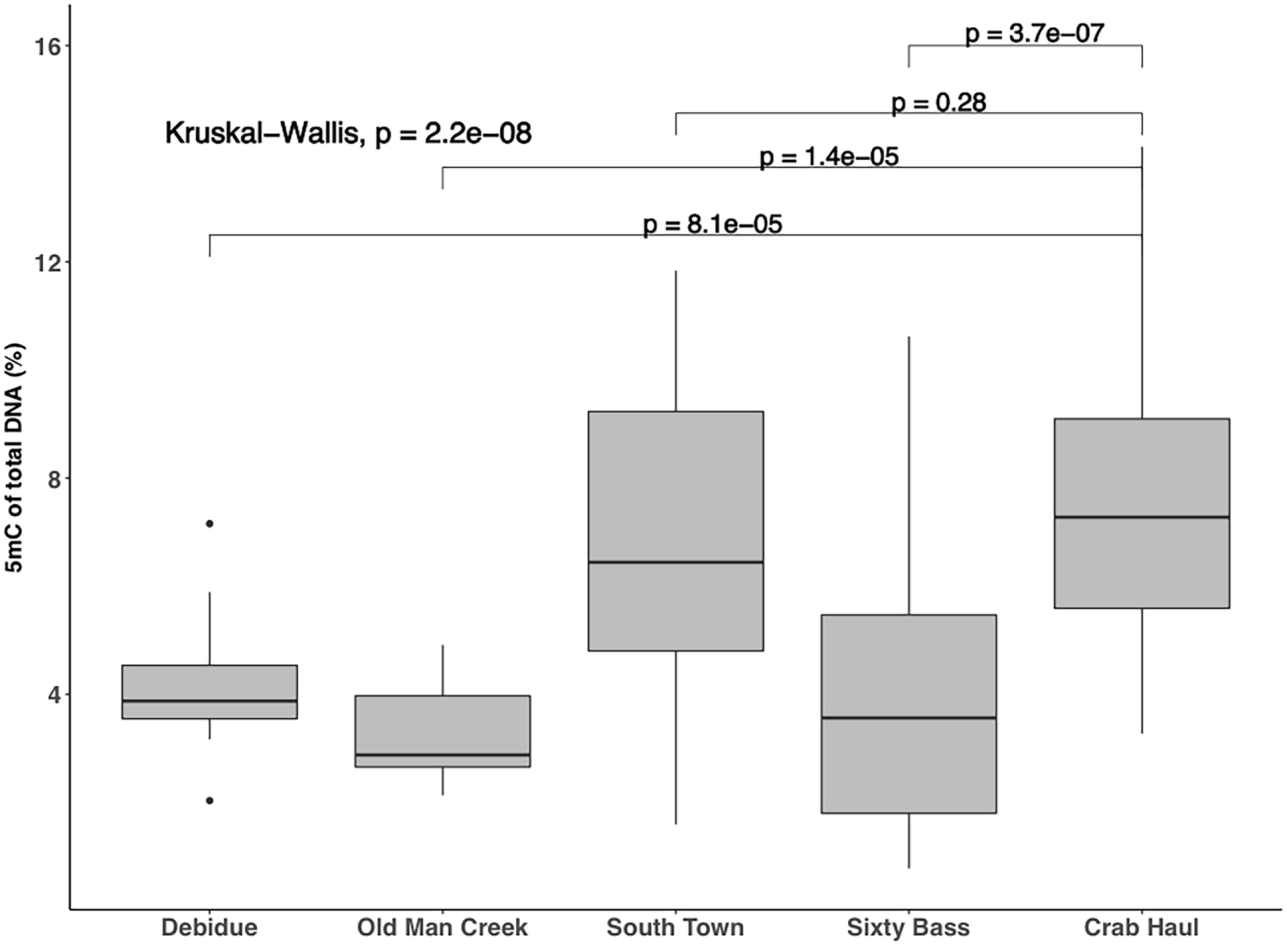
Variation of methylation among sites. Spatial variation of global DNA methylation among sampling sites was observed to be statically significant between Crab Haul and the Debidue, Old Man Creek, and Sixty Bass locations.

Overall, methylation of the *S*. *alterniflora* plants sampled across North Inlet was 5.76 ± .29% (mean ± SE, n = 109, Fig 2). Values ranged from 0.79–14.13% (interquartile range = 3.58–7.46%); in addition, the range differed at each sampling location (Fig 2). Heterogeneity of methylation levels among sites was statistically significant (χ^2^ = 41.434, *df* = 4, *p* << 0.0001, Kruskal-Wallis rank sum test). Post-hoc analysis indicated significant differences between Crab Haul and the three of the four locations sampled: Debidue, Old Man Creek, and Sixty Bass respectively (*t = 5*.*61*, *df* = 24.96, *p* << 0.0001; *t* = 8.26, *df* = 33.84, *p* << 0.0001; *t* = 6.08, *df* = 63.02, *p* << 0.0001, Games-Howell tests; Fig 3).

The CV of methylation values for the technical replicates of all plants sampled was 42.39. Mean CVs for technical replicates t different sites were comparatively small: South Town Asexual 8.22 ± 1.64, South Town Sexual 9.12 ± 1.39, Old Man Creek 16.11 ± 3.65, Health Marsh (Sixty Bass) 12.87 ± 4.97, AMD Periphery (Sixty Bass) 22 ± 3.28, AMD (Sixty Bass)16.6 ± 3.59, Crab Haul Creek 11.29 ± 1.54.

**Fig 4:**
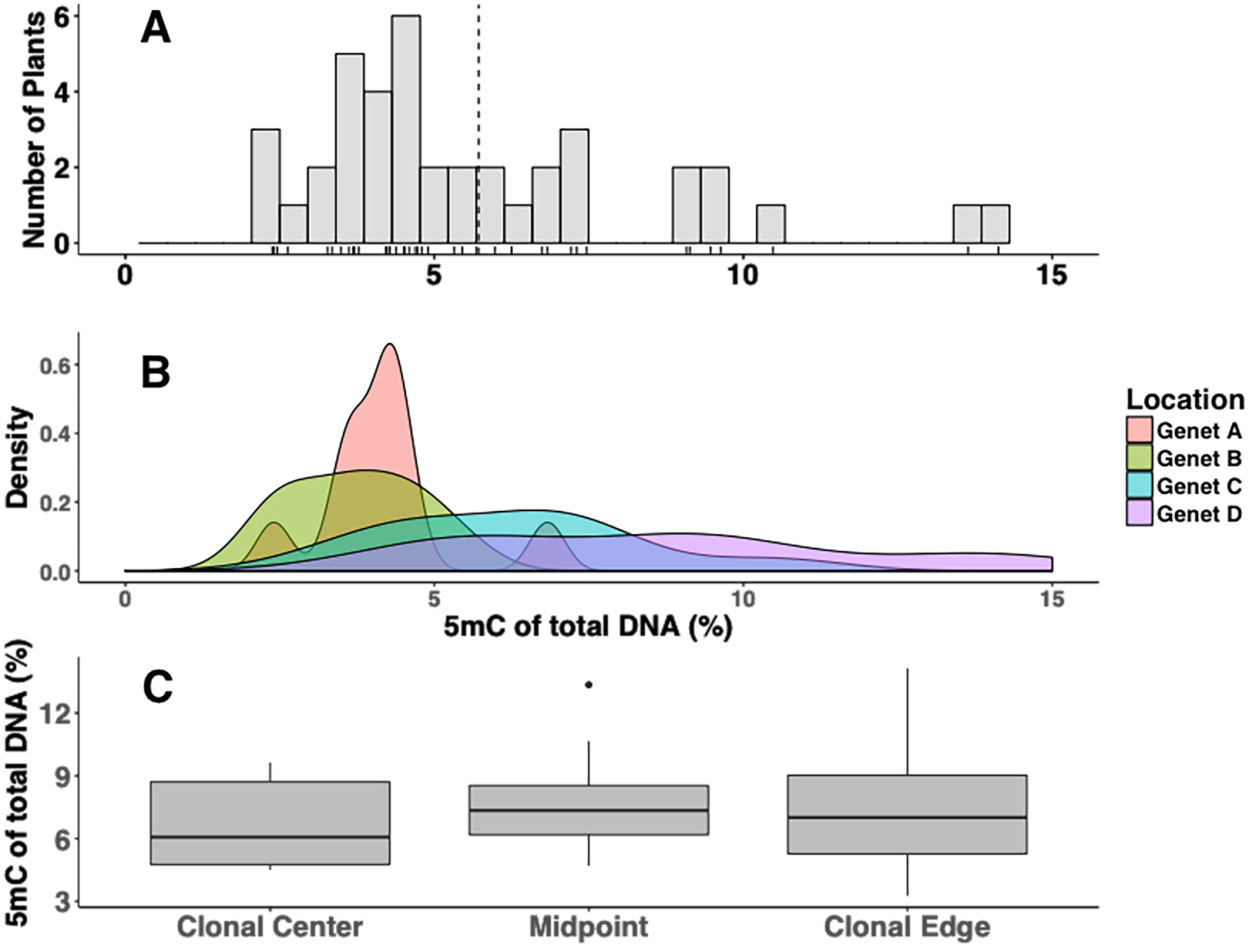
Methylation variation within and between discrete genets of *S*. *alterniflora* from the Crab Haul Creek site. **A**. Frequency distribution of 5mC observations pooled across genets. **B**. Frequency distributions of methylation for each of the four genets. **C**. Boxplots of methylation variation based on position within the genet structure.

At Crab Haul Creek, methylation of known distinct genets ranged from 2.37–14.13% with a grand mean of 5.71 ± 0.48% for the site (interquartile range of all observations = 3.76–6.93%, Fig 4, n = 40). There was significant heterogeneity of methylation levels among individual ramets within genets (χ^2^ = 21.518, *df* = 3, *p* << 0.0001, Kruskal-Wallis rank sum test). Within-clone variation of methylation was not significantly correlated to position within the genet (*ρ* = 0.1, *p* = 0.49, Spearman Correlation). The CV among all four genets’ samples was 33.45. The CV for technical replicates were as follows: Genet A 10.22 ± 1.65 (N/S), 8.21 ± 3.01(E/W); Genet B 10.44 ± 2.12 (N/S), 13.87 ± 3.62 (E/W); Genet C 12.81 ± 6.23 (N/S), 20.24 ± 7.52 (E/W) Genet D 7.42 ± 1.33 (N/S), 7.09 ± 1.77 (E/W).

**Fig 5:**
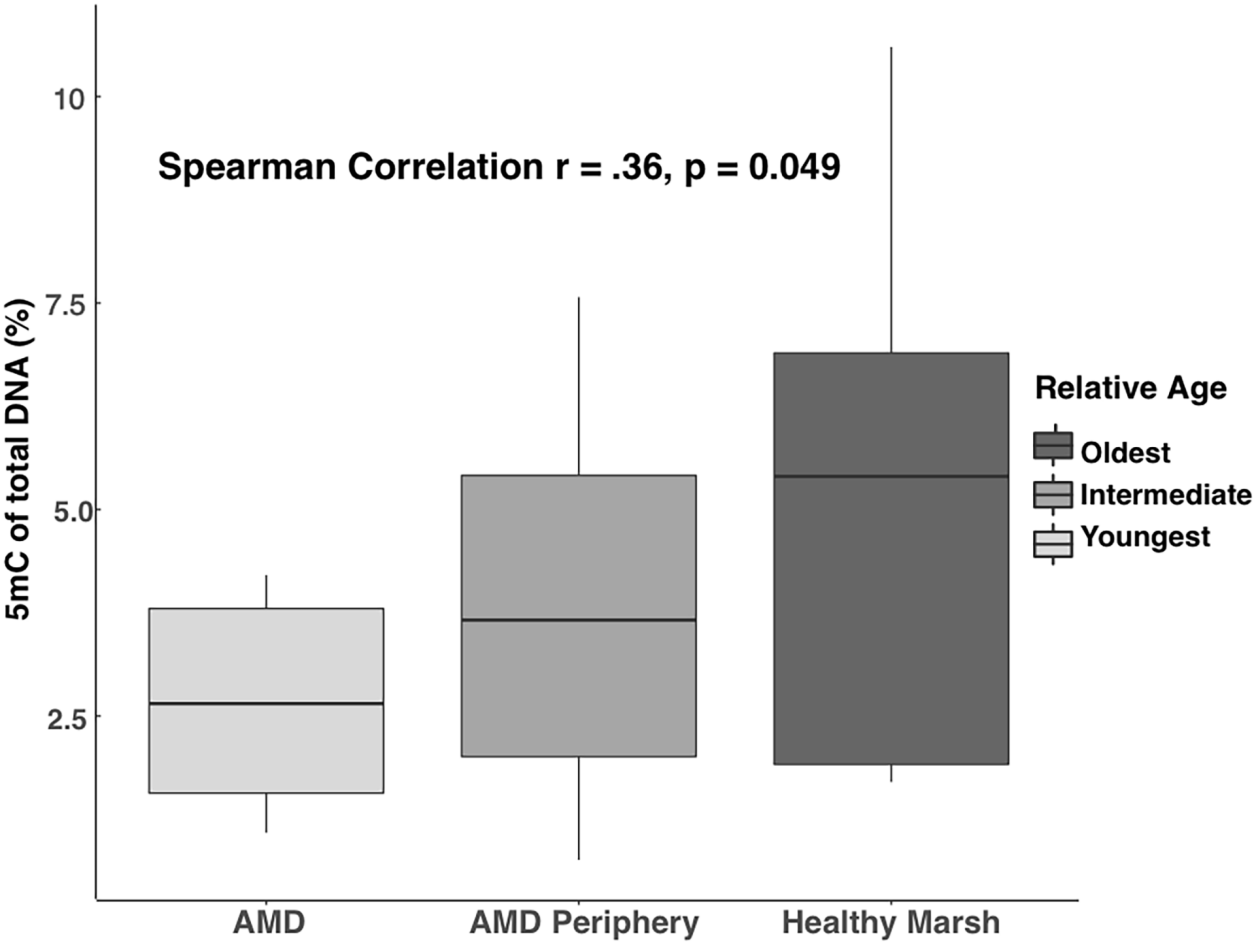
Boxplots of global cytosine methylation at the Sixty Bass site. Samples were taken from three distinct zones in 2014 following a full recolonization after an acute marsh dieback event in the Fall of 2001. Work conducted by Hughes et al. (2012) marked the extent of the dieback and subsequent regrowth/clonal expansion into the epicenter of the dieback.

Methylation from AMD, AMD Periphery, and Healthy Marsh samples at the Sixty Bass location ranged from 0.78–10.62% (interquarile range = 1.8–5.47%, n = 10 per category) with a grand mean of 3.9 ± 0.08% (Fig 5). A statistically significant positive correlation between increasing methylation and years since recolonization (*ρ* = 0.36, *p* = 0.049, Spearman Correlation) was observed. Healthy marsh plants are composed of ramets unaffected by the 2001 AMD and have continuously inhabited the Sixty Bass location. Conversely, the AMD Periphery and AMD plants are composed of ramets that have only fully recolonized the AMD area within the past 5 to 8 years.

**Fig 6:**
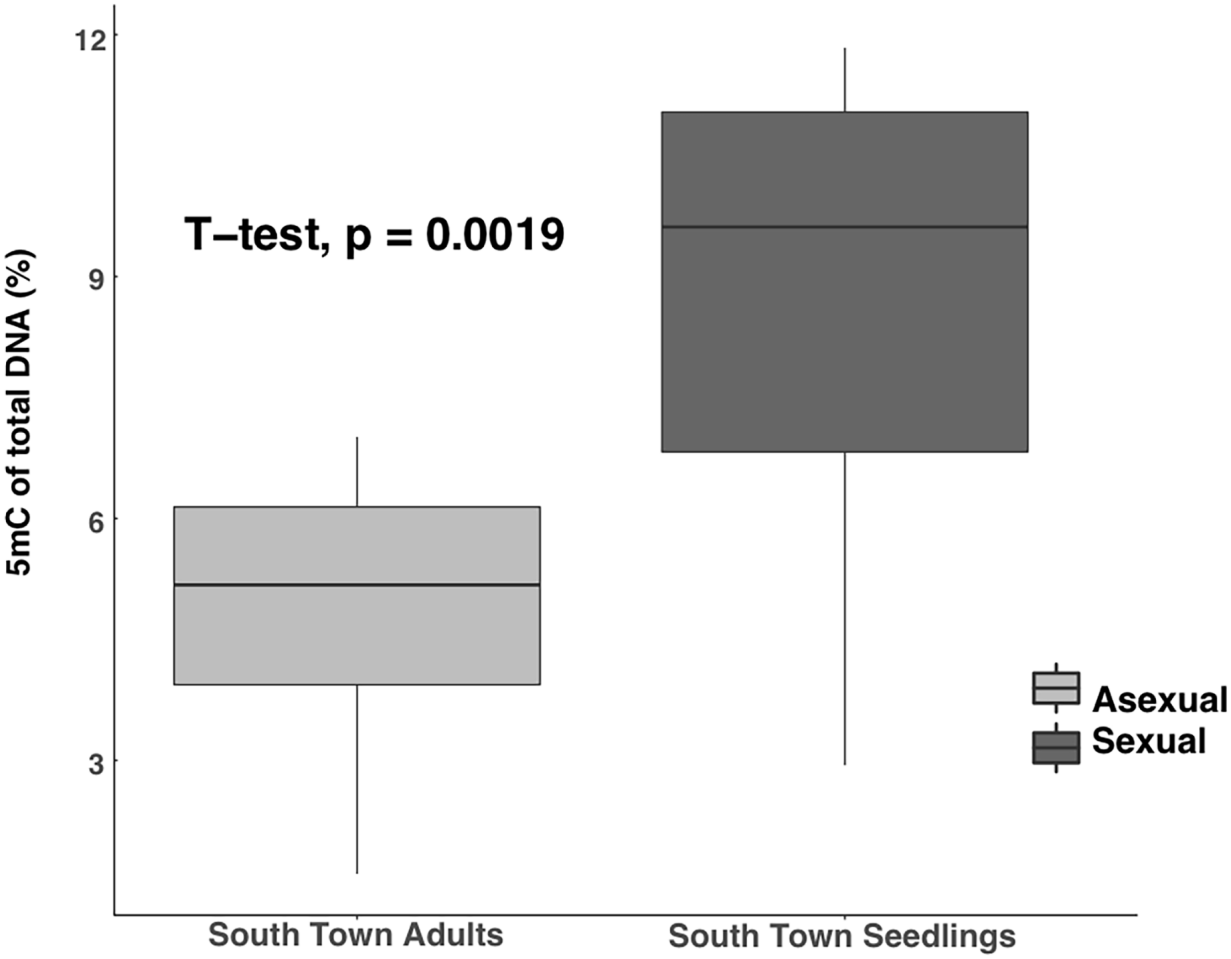
Boxplots of global cytosine methylation from the South Town site. Asexually produced adults had significantly lower methylation than the 3 to 4 month old sexually produced seedlings.

Samples from the South Town location included established asexually produced *S*. *alterniflora* plants in addition to sexually produced seedlings. Detected methylation within the South Town location ranged from 1.6–11.84% with a grand mean of 6.75 ± .16 (interquartile range = 4.8–9.23%, n = 20). The current study found significantly different methylation between the sexually produced seedlings (8.80 ± 0.92) and the asexually produced adults (4.70 ± 0.60) (*t* = -3.76, *df* = 18, *p* < 0.001, Fig 6).

## Discussion

The current study’s investigation into global DNA methylation across a natural populations of the halophyte *S*. *alterniflora* uncovered multiple axes of variation. Substantial variation of methylation was observed across sites, variation among genets, and developmental variation. Plants from an AMD site displayed a positive correlation between age and methylation. Conversely, recently produced sexual seedlings showed higher levels of methylation compared to the surrounding periphery of asexually produced adult plants at the South Town sampling site.

Variation of global methylation has been documented to have functional consequences including altered gene expression and genomic instability [21,50,51] and can be influenced by both genetic variation and local environmental conditions. Variation of DNA methylation among plants within the North Inlet basin mirrored similar findings seen within natural populations of Japanese knotweeds, orchids, and various other plants [32,52]. Previously reported by Messeguer et al. [53], mean global methylation within angiosperms ranged from 5–37%. This study’s results fell to the lower limits of the reported methylation and in some cases below previously reported values.

This study’s results show a significant difference among samples taken from the Crab Haul site and the Debidue, Old Man Creek, and Sixty Bass (Fig 3). As the most inland creek system of North Inlet, the Crab Haul Creek basin runs adjacent to forest-marsh upland with a large freshwater lens. The North-West boundary of Crab Haul Creek has a steep gradient with a short distance to local creek which could result in the flow of fresh water into basin, ultimately decreasing the salinity content. The decrease in salinity could be the contributing factor to the significant difference of methylation between the Crab Haul Creek site and Debidue, Old Man Creek, and Sixty Bass. Stress-induced increases and decreases in methylation have been documented in various plant species [54–57] including *S*. *alterniflora* [44]. In some cases, the alterations in methylation can be inherited [22,58]. Wang et al. [56] showed considerable variation of methylation both globally and within the promoter regions of salinity stress-response genes of wheat cultivars after salinity treatments. Thus, one possible contributor to among-site variation of methylation is variation of salinity stress.

Associations between decreasing global DNA methylation and aging have been reported in various studies [59–61]. The current study tested this association in three contexts: age variation within genets, age variation within a site, and seedlings versus adults. Age variation within genets and the association with methylation was tested with respect to each genet sampled from the Crab Haul site. Expansion of *S*. *alterniflora* genets occur via a circular spread pattern with newly produced ramets composing the outer most edges. Proffitt et al. [62] described clonal expansion as a continuum originating from a clonal center in which ramets are produced via underground rhizomes in either densely clustered or widely spaced patterns. Therefore, the clonal center would be composed of the oldest founding individuals while the composition of outer sections would be made up of younger ramets. The current study did not find any significant associations between position and methylation within genets, possibly due to the high level of methylation heterogeneity. Errors could have been introduced by nonsymmetrical clonal expansion in addition to sampling relatively young aged genets. In order to identify discrete genets, sampling was restricted to relatively young and more recently established genets. The difference in age between the clonal centers and edges would therefore only be approximately 3–4 years which may not been enough time to detect significant changes in methylation. Approximation of the clonal center was based on the intersection of two transects and assumed an equilateral expansion out of ramets away from the center as the genet grows.

Acute and large scale dieback events have been heavily studied, particularly after 100,000 ha of *S*. *alterniflora* in the Mississippi River deltaic plain were affected in the year 2000 [63–66]. Presently there is much dispute regarding the cause of the dieback event in Louisiana, but environmental stress is one of the acknowledged constants across sites. Previous studies completed by Hughes et al. [47] at the Sixty Bass location could not conclusively identify an environmental stressor that preceded the AMD event from the year 2001 at North Inlet. They proposed that the mostly like trigger for the event was an increase in salinity which overwhelmed the osmoregulation capacity of *S*. *alterniflora*. This study observed a significantly positive correlation between presumptive age since recolonization and methylation, indicating a trend of methylation increasing with age. As noted above, salinity stress has the potential to generate both hypo- and hyper-methylation shifts across the genome, including within the promoter regions of salinity stress-response genes [56]. It is possible that *S*. *alterniflora* plants recolonizing the AMD epicenter could have decreased global methylation levels in response to hypersaline conditions. This is consistent with the among site variation we observed with Crab Haul Creek, the low salinity site, having the highest global methylation.

The South Town sampling site was primarily composed of a periphery of densely clustered clonal plants surrounding an interior of mudflat comprised of wrack and recently (within a few months) emerged sexually produced seedlings. During the process of sexual reproduction, both embryogenesis and gametogenesis display large amounts of epigenetic resetting and alteration across the genome [17,18]. Well-documented in mammals, epigenetic resetting in both primordial germ cells and the early embryo serve as mechanism to prevent the accumulation of epigenetic marks from one generation to another [67, 68]. In contrast to mammals, plants do not undergo a complete epigenetic resetting event but rather experience several partial resetting events. Incomplete or partial epigenetic resetting of the genome can allow for transgenerational inheritance of modifications like DNA methylation of transposable elements [69,70] in addition to genome-wide hypomethylation in female gametes which initiates RNA-directed DNA methylation [18]. Our results show a significant difference in the amount of methylation between known asexually-produced ramets and sexually-produced seedlings. This difference could be driven by the age or mode of reproduction of *S*. *alterniflora* individuals. High methylation levels in both the Sixty Bass healthy marsh and South Town seedling suggest that the reproductive mode rather than age is more likely. Alternatively, the level of genotypic diversity among the seedlings and clonal populations could explain the differences in methylation levels. Genotypes containing higher percentages of cytosines have the potential to increase global methylation across the genome.

In conclusion, the present study has quantified the variation of cytosine methylation in a natural population of *S*. *alterniflora* within North Inlet. Genets across the basin display both within and among clonal epigenetic heterogeneity similar to that observed within many plant species [38]. Global cytosine methylation detected within the genet structure displays similar levels of variation as among genet methylation. We found a significantly positive correlation between cytosine methylation and presumptive age at the Sixty Bass location, which experienced an AMD. Significant differences in cytosine methylation were detected between asexually produced adult individuals and sexually produced seedlings at the South Town sampling location. This study provides further insight into the epigenetic variation of a natural population among sites, among clones, within clones, in the aftermath of AMD, and between modes of reproduction.

## Supporting information

S1 Dataset Methylation values from North Inlet *S*. *alterniflora* samples. (XLSX)
